# Firesetting in Childhood and Adolescence

**DOI:** 10.3389/fpubh.2013.00040

**Published:** 2013-10-08

**Authors:** Joav Merrick, Carrie Howell Bowling, Hatim A. Omar

**Affiliations:** ^1^National Institute of Child Health and Human Development, Jerusalem, Israel; ^2^Health Services, Division for Intellectual and Developmental Disabilities, Ministry of Social Affairs and Social Services, Jerusalem, Israel; ^3^Division of Pediatrics, Hadassah Hebrew University Medical Center, Jerusalem, Israel; ^4^Division of Adolescent Medicine, Kentucky Children’s Hospital, University of Kentucky, Lexington, KY, USA; ^5^Lexington Fire Department, Fire Investigation Bureau, Lexington, USA

**Keywords:** pyromania, fire, adolescent behavior, firesetting behavior, childhood

## Introduction

Fire is the rapid oxidation of a material in the exothermic chemical process of combustion, releasing heat, light, and various reaction products. Fire is intriguing and therefore something that will attract a curious child from an early age.

In 2010, the United States fire department responded to 44,900 fires started by someone, usually a child, playing with fire. These fires caused 90 civilian deaths, 890 civilian injuries, and $210 million in direct property damage (1). Preschoolers and kindergartners are most likely to start these fires, while playing with matches or lighters and most likely to die in these fires. Most fire-play fires (77%) started outside, but most associated deaths (97%) were in home structure fires. Almost half (46%) of people who start reported home fires by playing were 5 years old or younger. Two out of five (40%) child-playing home structure fires began in the bedroom. Mattresses and bedding were the items first ignited in 24% of child-playing home structure fires and 29% of associated civilian fire deaths (1).

## Fire-Setter

In general, a fire-setter is any individual who sets a fire for various reasons. Accidental or curiosity fire-setting is defined as fire-starting behavior often by unsupervised children (usually age 5–10 years of age) with access to matches or lighters. In order to prevent and make children aware of the danger of fires many fire departments and educational agencies have teamed up and created teaching and educational material for children starting already in early childhood education (2, 3).

The United States Fire Administration has developed a very comprehensive juvenile fire-setter intervention handbook (3) that describes in great details the juvenile fire-setter and their families, intervention, community work, and how to build a juvenile fire-setter program (3).

In spite of this handbook there is still no state or nationwide data base on firesetting in the United States (4). One retrospective study from Ohio reviewed charts of all fire setters between the years 2003–2005. There were 133 participants aged 3–17 years and analysis of the data set found 26% of the peak ages for fire involvement to be 12 and 14 years. Location, ignition source, and court ordered participants were divided into two age groups: 3–10 years (*N* = 58) and 11–17 years (*N* = 75). Bedrooms ranked first for the younger population and schools for the latter. Fifty-four percentage of the 133 participants used lighters over matches. Twelve percentage of the 3- to 10-year-olds were court mandated, compared with 52% of the 11- to 17-year-olds. Recidivism rates were 4–10% with a 33–38% survey return rate.

## Pyromania

Whereas a child fire-setter is usually curious about fire and has the desire to learn more about fire, a pyromaniac is more than just a simple fire-setter, he or she is one who has an unusually bizarre impulse or desire to set intentional fires. Pathological fire-setting, pyromania, is when the desire to set fires is repetitive and destructive to other people or property. Pyromania is a deliberate, planned, and persistent behavior.

One recent study (5) examined the prevalence and correlates of intentional fire-setting behavior in the United States from a nationally representative sample of United States residents 18 years and older. Structured psychiatric interviews (*N* = 43,093) were completed by trained lay interviewers between 2001 and 2002. Fire-setting as well as mood, anxiety, substance use, and personality disorders were assessed and the prevalence of lifetime fire-setting in the United States population was 1.0%. Males, white, 18–35 years old, born in the United States, and living in the western region of the United States had significantly higher rates of fire-setting. Fire-setting was significantly associated with a wide range of antisocial behaviors and analyses identified strong associations between lifetime alcohol and marijuana use disorders, conduct disorder, antisocial and obsessive-compulsive personality disorders, and family history of antisocial behavior.

Intentional illicit fire-setting behavior was associated with a broad array of antisocial behaviors and psychiatric comorbidities (5).

## Research

The research of firesetting has been conducted in different ways and lack a coherent, consistent, and comprehensive set of empirical findings (6). A recent review (6) concluded that despite a number of risk factors being repeatedly identified, an understanding of the etiology behind firesetting behavior and potential developmental trajectories remains theoretically rather than empirically based (6). Existing theories do not take sufficient account of the complexities of firesetting behavior and there is not yet a typology and accompanying assessment that has undergone thorough empirical testing and is of significant clinical utility.

Due to the relationship between firesetting and antisocial behavior there is a need for interdisciplinary intervention for fire-setters that includes assessment and provides an individualized and developmentally appropriate approach.
